# Supplementary material for: Twist and Snout: Head and Body Morphologies Determine Feeding Kinematics in Substrate-Biting Fishes
Source: Integr Org Biol. 2025 Jul 25;7(1):obaf032. doi: 10.1093/iob/obaf032 (PMC12392097; doi:10.1093/iob/obaf032)
Supplement: obaf032_Supplemental_Files [file obaf032_supplemental_files.zip › French abstract.docx]

Résumé

Chez les téléostéens, le mécanisme d’alimentation consistant à mordre des proies fixées au substrat a évolué à plusieurs reprises et est associé à des convergences morphologiques telles qu’un corps profond et une tête allongée et effilée. Toutefois, le rôle fonctionnel de ces morphologies chez les poissons qui mordent le substrat reste encore à établir. Nous avons ici testé l'hypothèse selon laquelle ces morphologies fonctionnent comme des surfaces de contrôle affectant la cinématique d'alimentation lors de la morsure. Pour ce faire, nous avons développé des modèles physiques simplifiés d’espèces de poissons de récif connues pour utiliser la morsure du substrat comme mécanisme d’alimentation. Ces maquettes expérimentales nous ont permis d’examiner le rôle de la morphologie de la tête, du corps et des nageoires dans la cinématique d'alimentation, en particulier dans le cadre de l’extraction de proies fixées au substrat. Ces modèles ont simulé le mouvement latéral rapide de la tête ; mouvement déjà documenté chez les espèces qui se nourrissent d’algues fixées au substrat par morsure. À l'aide de ces modèles qui reflètent la variation morphologique naturelle de poissons qui mordent le substrat, nous avons testé (i) l'influence de différentes morphologies de la tête sur la vitesse de déplacement de celle-ci, et (ii) l'effet de différentes morphologies du corps sur la stabilité du corps pendant les mouvements de la tête. Nous avons ainsi montré que le moment d’inertie (MOI) de la tête et le MOI du corps expliquent la majorité des variations de la vitesse de la tête et du déplacement du corps, respectivement. Une diminution du MOI de la tête a entraîné des mouvements latéraux plus rapides de celle-ci, facilitant ainsi l’extraction des proies fixées au substrat. Une augmentation du MOI du corps, relative à celui de la tête, a minimisé le déplacement latéral du corps lors des morsures, et ainsi amélioré la stabilité. Globalement, nos résultats suggèrent que les corps comprimés latéralement et les museaux effilés fonctionnent comme des surfaces de contrôle chez les poissons qui mordent le substrat. Nous proposons qu'une pression sélective favorisant l’expansion de la surface latérale sous-tend la convergence morphologique dominante observée chez les poissons de récif qui recourent à la morsure pour se nourrir.
