# Supplementary material for: Twist and Snout: Head and Body Morphologies Determine Feeding Kinematics in Substrate-Biting Fishes
Source: Integr Org Biol. 2025 Jul 25;7(1):obaf032. doi: 10.1093/iob/obaf032 (PMC12392097; doi:10.1093/iob/obaf032)
Supplement: obaf032_Supplemental_Files [file obaf032_supplemental_files.zip › Table S1.docx]

| Family | Species | Primary Diet | FishBase photo |
| --- | --- | --- | --- |
| Chaetodontidae | *Chaetodon auriga* | Facultative corallivore | Chaur_u7 |
| Chaetodontidae | *Chaetodon melapterus* | Corallivore | Chmel_ua |
| Pomacanthidae | *Centropyge multispinis* | Invertivore | Cemul_u7 |
| Pomacanthidae | *Pomacanthus imperator* | Invertivore | Poimp_u6 |
| Labridae | *Anampses caeruleopunctatus* | Invertivore | Ancae_u2 |
| Labridae | *Iniistius pavo* | Invertivore | Xypav_m1 |
| Labridae | *Calotomus viridescens* | Herbivore | Cavir_m3 |
| Acanthuridae | *Zebrasoma xanthurum* | Herbivore | Zexan_u1 |
| Acanthuridae | *Acanthurus fowleri* | Herbivore | Acfow_u0 |
| Acanthuridae | *Acanthurus nigrofuscus* | Herbivore | Acnig_ua |
| Acanthuridae | *Naso unicornis* | Herbivore | Nauni_u1 |
| Siganidae | *Siganus vulpinus* | Herbivore | Sivul_u2 |
| Siganidae | *Siganus rivulatus* | Herbivore | Siriv_u3 |
| Pomacentridae | *Stegastes lacrymatus* | Herbivore | Pllac_u0 |
| Pomacentridae | *Pomacentrus aquilus* | Herbivore | Poaqu_u6 |
| Kyphosidae | *Kyphosus vaigiensis* | Herbivore | Kycin_u3 |
| Kyphosidae | *Microcanthus strigatus* | Herbivore | Mistr_u2 |

Supplementary Table. Model species info
